# Pyriproxyfen Disrupts the Ongoing Spermatogenesis Wave in *Danio rerio* Potentially Mediated by Voltage‐Dependent Calcium Channels and Protein Kinase C

**DOI:** 10.1002/jat.4801

**Published:** 2025-05-01

**Authors:** Bruna Antunes Zaniboni, Vanessa Staldoni de Oliveira, Gabriel Adan Araujo Leite, Valdelúcia Maria Alves de Souza Grinevicius, Rozangela Curi Pedrosa, Fátima Regina Mena Barreto Silva

**Affiliations:** ^1^ Instituto de Bioeletricidade Celular (IBIOCEL): Ciência & Saúde Universidade Federal de Santa Catarina Florianópolis SC Brazil; ^2^ Departamento de Bioquímica, Centro de Ciências Biológicas Universidade Federal de Santa Catarina Florianópolis SC Brazil; ^3^ Departamento de Biologia Celular, Embriologia e Genética, Centro de Ciências Biológicas Universidade Federal de Santa Catarina Florianópolis SC Brazil

**Keywords:** calcium channel, in silico assays, infertility, Larvicide, protein kinase C, spermatogenesis

## Abstract

Pyriproxyfen (PPX) is an analog of the juvenile hormone from insects. Following our previous studies, for the ex vivo short‐term effect, we chose 10^−9^ M pyriproxyfen to analyze the morphology of spermatogenesis wave cells. In silico docking and ADMET (Absorption, Distribution, Metabolism, Excretion, Toxicity) studies were carried out to preliminarily predict possible interaction modes between PPX and the T‐type voltage‐dependent calcium channel (T‐VDCC), as well as with protein kinase C (PKC), as we previously reported by using pharmacological approach. The in silico ADMET evaluations revealed that PPX demonstrates notable lipophilicity. Moreover, PPX is predicted to inhibit the enzymatic activity of CYP1A2, CYP2C19, CYP2C9, and CYP2D6. Furthermore, in silico molecular docking analyses revealed that PPX has the potential to interact with the T‐VDCC through hydrogen bonds with Gln1653 and hydrophobic interactions with Leu291, Phe322, Phe1607, and Leu1656. Possible interactions of PPX with PKC involve ionic bonding with Lys463, hydrogen bonds with His592, and hydrophobic interactions with Lys463, Val596, Gly591, Phe593, Lys611, Asp711, and Leu714 reinforcing these both targets to PPX. In summary, short‐term PPX exposure influenced the morphology of testicular cells (spermatids, spermatozoa, and Leydig cells) through interactions with molecular targets. Findings reveal the bimodal effects (on morphology and signaling) of this compound on specific cells within the spermatogenic wave, endocrine cells, and signal transduction proteins. This interference may impair reproduction and lead to male infertility. In addition, the prediction from both molecular docking and ADMET supported our in vitro mechanistic analysis firstly reported in the testis of *Danio rerio* .

## Abstract 2

Pyriproxyfen (PPX) alters the morphology of testicular cells in *Danio rerio* and may impair male fertility. In silico and in vitro analyses revealed interactions of PPX with T‐type calcium channels and PKC, supported by docking and ADMET predictions. PPX showed high lipophilicity and potential to inhibit key CYP enzymes. Short‐term exposure affected spermatids, spermatozoa, and Leydig cells, indicating bimodal effects on cell morphology and signaling pathways involved in spermatogenesis.

## 1 Introduction

Although pyriproxyfen (PPX) is primarily used to control *Aedes aegypti* mosquitoes, its widespread application has raised concerns due to its unintended effects on nontarget organisms and ecosystems. It is among the insecticides evaluated by the World Health Organization (WHO) and recommended for inclusion in drinking water (0.01 mg/L) as a preventive measure against mosquito‐borne diseases such as dengue, chikungunya, and Zika (WHO 2007). In Brazil, it has been used since 2014 by the National Dengue Control Program of the Ministry of Health and is also widely used in agriculture on crops such as beans, tomatoes, soybeans, grapes, and coffee as recommended by the Ministry of Agriculture and Livestock (ADAPAR 2021). PPX functions by mimicking the juvenile hormone in insects, disrupting metamorphosis and reproduction (Sullivan and Goh 2008). However, several studies have reported its toxicity in nontarget organisms, including hepatocyte apoptosis and cellular damage in rats (Liu et al. 2020), neurotransmitter imbalances in *Oreochromis niloticus* (Da Silva et al. 2020) and *Aporrectodea caliginosa* (Nasr and Badawy 2015), increased oxidative stress and ovarian follicular atresia in *Danio rerio* (Oliveira et al. 2023), and reduced offspring production in *Daphnia magna* (Olmstead and LeBlanc 2003), among other documented adverse effects (Maharajan et al. 2020; Oliveira et al. 2021; Silva et al. 2024).

The testis of *D. rerio* , commonly known as the zebrafish, consists of testicular lobes containing seminiferous tubules, where spermatogenesis occurs. Sertoli cells surround these tubules, providing structural support and nutrients to develop germ cells, while Leydig cells, located between the tubules, produce androgens essential for sperm maturation. In fish, spermatogenesis follows a cystic pattern, with Sertoli cells forming cysts that enclose synchronously developing germ cells, enhancing spermatogenic efficiency and reducing germ cell apoptosis compared with higher vertebrates (Schulz et al. 2010). The spatial organization of *D. rerio* testes varies, exhibiting either restricted or unrestricted spermatogonia distribution. Diploid spermatogonia differentiate into mature sperm daily, a process maintained by spermatogonial stem cells capable of self‐renewal and commitment to sperm development (Leal et al. 2009). Furthermore, chromatin condensation in sperm, regulated by factors such as calcium, is crucial for generating a compact and hydrodynamic form, protecting the parental genome from physical and chemical damage (Golpour et al. 2017). Throughout the process of spermatogenesis, calcium plays a fundamental role as an intracellular messenger, being essential for sperm motility, fertilization, cell differentiation, and the acrosomal reaction of sperm. However, high concentrations of intracellular calcium can lead to apoptosis, highlighting the importance of calcium homeostasis for cellular function (Golpour et al. 2017).

The hypothesis of endocrine disruption suggests that natural and synthetic substances in the environment can modulate or interfere with endocrine signaling, ultimately affecting development and reproduction in vertebrates, including *D. rerio* (Segner 2009). *D. rerio* has emerged as a valuable vertebrate model for studying the effects of endocrine‐disrupting compounds (EDCs) due to its well‐characterized reproductive system, rapid spermatogenesis cycle, and sensitivity to environmental changes. Unlike mammals, in which spermatogenesis takes approximately 45 days, *D. rerio* completes a full wave of spermatogenesis in just 6 days, making it particularly advantageous for tracking the progression of germ cell development and assessing the impact of toxicants over short experimental periods (Abid et al. 2014; Batista‐Silva et al. 2020a; Leal et al. 2009).

Beyond its efficiency in reproductive studies, *D. rerio* share key molecular mechanisms and hormonal signaling pathways with higher vertebrates, including humans, making it a highly translational model for investigating calcium homeostasis, endocrine disruption, and testicular function (Segner 2009). This similarity allows researchers to explore fundamental aspects of spermatogenesis and its regulation under the influence of toxicants, such as PPX, while drawing insights applicable to broader vertebrate reproductive biology. Furthermore, *D. rerio* are particularly responsive to EDC exposure, as demonstrated by previous studies on methylparaben (MeP), another known endocrine disruptor, which significantly inhibited spermatogenesis by increasing the proportion of early‐stage germ cells (spermatogonia and spermatocytes) while reducing mature sperm production at environmentally relevant concentrations (Hu et al. 2023). Such findings reinforce the relevance of *D. rerio* as a robust model for evaluating the reproductive toxicity of various contaminants, including PPX.

Previous research has demonstrated that PPX induces an increase in testicular intracellular calcium levels in *D. rerio* (Oliveira et al. 2021). Intracellular calcium is tightly regulated and maintained at low cytosolic concentrations; thus, any influx significantly alters its basal levels, acting as a critical second messenger in various signaling pathways (Szydlowska and Tymianski 2010).

The study by Oliveira et al. (2021) investigated this effect using inhibitors and activators of ion channels, pumps, exchangers, protein kinases, and calcium chelators, which were added 15 min before the introduction of ^45^Ca^2+^ and PPX. Their findings demonstrated that PPX increases ^45^Ca^2+^ influx through multiple mechanisms, including the opening of T‐type voltage‐dependent calcium channel (T‐VDCC), inhibition of sarco/endoplasmic reticulum Ca^2+^‐ATPase (SERCA) and the sodium‐calcium exchanger (NCX, forward mode), and mobilization of calcium from intracellular stores. Additionally, they confirmed the involvement of potassium channels and protein kinase C (PKC) in PPX‐induced intracellular calcium elevation. Notably, pre‐incubation with a PKC inhibitor completely blocked the PPX‐induced ^45^Ca^2+^ influx, reinforcing the role of PKC in this signaling cascade.

Building upon these findings, the present study further examined morphometric changes in testicular cells following PPX exposure (10^−9^ M for 7 days) and employed in silico approaches, including molecular docking and ADMET predictions, to explore PPX interactions with PKC and T‐VDCC.

The T‐VDCC, when open, can promote the entry of ions at a high flow of 10 million ions per second, which can stimulate intracellular signaling mediated by toxicants (Batista‐Silva et al. 2020a, 2020b; Golpour et al. 2017; Lacinová 2005). In addition, high calcium influx can trigger exocytosis pathways and may activate key enzymes of metabolism (Do Nascimento et al. 2020; Zanatta et al. 2011, 2013). PKC is a pivotal protein in signal transduction that is activated by calcium and diacylglycerol (DAG). When activated, it attaches to the membrane and phosphorylates other proteins that have the amino acid residues Ser and Thr in a specific sequence (Boileve et al. 2024; Spitaler and Cantrell 2004). In addition, phosphorylation occurs in signaling pathways that may involve cytoskeletal dynamic and/or nuclear proteins that regulate the gene expression of enzymes (Li et al. 2023; Spitaler and Cantrell 2004). Following up on the study by Oliveira et al. (2021), the objective of the present investigation was to elucidate the influence of 7 days of in vivo PPX exposure on the morphology of testicular cells (germ and somatic cells) of *D. rerio* . Additionally, we studied the potentials chemical interactions between PPX and T‐VDCC and the downstream signaling protein, PKC, which are potential targets of pesticide toxicity in the spermatogenic process, as demonstrated by Oliveira et al. (2021).

## 2 Materials and Methods

### 2.1 Chemicals

Pyriproxyfen (PPX), 4‐phenoxyphenyl (RS)‐2‐(2‐pyridylloxy) propyl ether, hematoxylin–eosin and paraffin were from Sigma Chemical Co. (St. Louis, MO, USA). All other reagents used for buffer preparation were of analytical grade.

### 2.2 Animals

Adult wild‐type strain male *D. rerio* weighing about 200–300 mg and measuring 2.5–3.5 were obtained from a commercial source (Acqua House, São José, SC, Brazil). Fish were acclimatized for 7 days before PPX exposure in a room with controlled temperature (21 ± 1°C), a 12/12 h light/dark cycle and housed in aquaria with dechlorinated water at pH 7.4, salinity 0.8 ± 1 g/L, and at 27 ± 1°C. Fish food (D‐50 Plus Granulat/Tropical, Florianópolis, SC, Brazil) was available twice a day and maintained according to ethical recommendations and following a protocol approved by the Brazilian College of Animal Experimentation and by the institutional Ethical Committee for animal Use (Protocol number UFSC/CEUA/PP00968).

### 2.3 Morphometric Analyses

For the preparation of samples for morphometric analysis, testes of five to six fish from both the control and 10^−9^ M PPX in vivo treatment groups were fixed in a modified Davidson's solution (Latendresse et al. 2002) at room temperature for 24 h. The ethanol dehydrated samples were included in paraffin and serial sections (4 mm thickness) were prepared. Three slides with three to six sections per slide were obtained for each specimen. The slides were stained with hematoxylin–eosin and examined using an Axio Scan scanner (Zeiss, Oberkochen, Germany). The sample sections were used to measure the nucleus area of testicular cells (spermatozoa, pale/dark spermatogonia, spermatids, primary spermatocytes, Leydig cells and Sertoli cells) from the control and PPX groups using the ZEN Lite 2012 SP2 program (version 1.1).

### 2.4 Statistical Analysis

Data are presented as means ± SEM of three independent experiments with, at least, three fish in each group per experiment. Statistical analysis was performed with Graph Pad Prism5, using Student's *t* test and one‐way ANOVA. The Bonferroni post hoc test was used to determine significant differences when *p* ≤ 0.05.

### 2.5 ADMET Prediction

The ADMET profile of a compound provides important information about its absorption, distribution, metabolism, physicochemical properties, excretion, and toxicity characteristics. These parameters will indicate how the compound behaves in the organisms studied. The SwissADME (http://www.swissadme.ch/index.php), ADMETLab (http://admet.scbdd.com), and admetSAR (http://lmmd.ecust.edu.cn/admetsar2) platforms were used to calculate these properties for PPX, using information from the literature. The canonical SMILES (Simplified Molecular Input Line Entry Specification) from PubChem (CID 91753) of PPX (CC (COC1 = CC=C(C=C1) OC2 = CC=CC=C2)OC3 = CC=CC=N3) was inserted in the analysis fields of the different tools.

### 2.6 T‐VDCC and PKC 3D Structures

The T‐VDCC structure of *D. rerio* (Uniprot entry A0A8M3AY82) was excluded from molecular docking analyses due to its structural incompleteness, which made it unsuitable for in silico simulations. Consequently, the 3D structure of the human T‐VDCC was selected instead. This structure, resolved through electron microscopy, is available in the Protein Data Bank (PDB) under the accession code, 7WLI (Berman et al. 2000), and is also referenced in the Uniprot database (Q9P0X4) (Holliday et al. 2015). The choice was justified by the conserved sequence and structural similarities between the human and *D. rerio* T‐VDCC proteins, as revealed through sequence alignment verified by a standard protein BLAST analysis and structural superimposition using Chimera tools.

The PKC from *D. rerio* used in this study was taken from the AlphaFold Protein Structure Database using the code AF‐A4QNX9‐F1‐v4, which provided the 3D‐folded protein file (.pdb format), calculated by AlphaFold algorithm (Guo et al. 2022; Jumper et al. 2021).

### 2.7 Homology Modeling and Structural Quality Assessment

The structure of T‐VDCC contains some unknown and low‐quality regions. Therefore, homology modeling was performed, resulting in a higher quality structure. A protein model, generated by Swiss homology modeling, was used for docking simulations.

The analysis of the quality of each protein 3D structure was carried out using the Ramachandran plot (Ramachandran et al. 1963) and Swiss Model Qualitative Model Energy Analysis (QMEAN) (Benkert et al. 2011). Simulations were conducted after the energy of the protein 3D structure was minimized using the Dock prep tool available in UCSF Chimera (Pettersen et al. 2004) (with Amber ff99bsc0 force field).

### 2.8 Ligand Structure

The 3D structure of the ligand was downloaded from Pubchem (CID 91753) in .sdf format. From there, its conversion into .mol2 format was done using UCSF Chimera (Pettersen et al. 2004), and the structure was then minimized using OpenBabel before being used for the molecular docking simulations.

### 2.9 Molecular Docking Analysis

The molecular docking simulations were performed using Autodock Vina (Pettersen et al. 2004) within UCSF Chimera (version 1.17.3). Visualization of chemical interactions inside the protein–ligand complex was performed with PyMOL (version 1.8.x Open Source) (Schrodinger 2015), LigPlot+ version 2.2 (Laskowski and Swindells 2011), and some tools available at the Proteins *Plus* server. All docking predictions used the standard parameters, according to Trott and Olson (2010), and the simulations were carried out in triplicate. The lowest scored‐energy positions files (.pdbqt format) were converted to .pdb with PyMOL and then visualized and analyzed.

## 3 Results

### 3.1 Morphometry of *D. rerio* Testicular Cells

**FIGURE 1:**
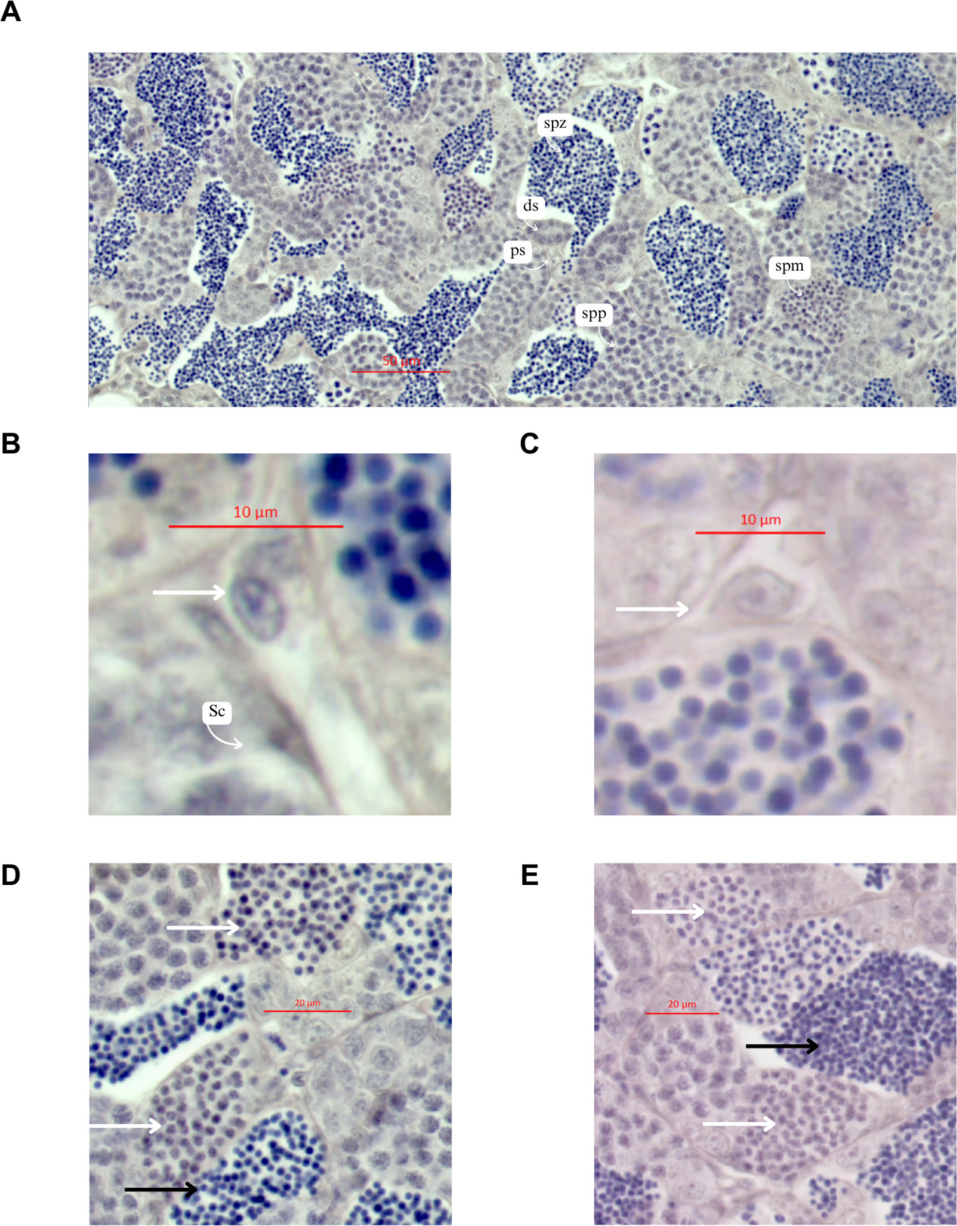
Optical microscopy of the *Danio rerio* testis. Control group with the presence of pale spermatogonia (ps), dark spermatogonia (ds), primary spermatocytes (spp), spermatids (spm) and spermatozoa (spz) (A); (B,C) Leydig cell and Sertoli cell nuclei (Sc) in control and PPX‐treated groups, respectively; (D,E) spermatid nuclei in control and PPX‐treated groups, respectively.

**FIGURE 2:**
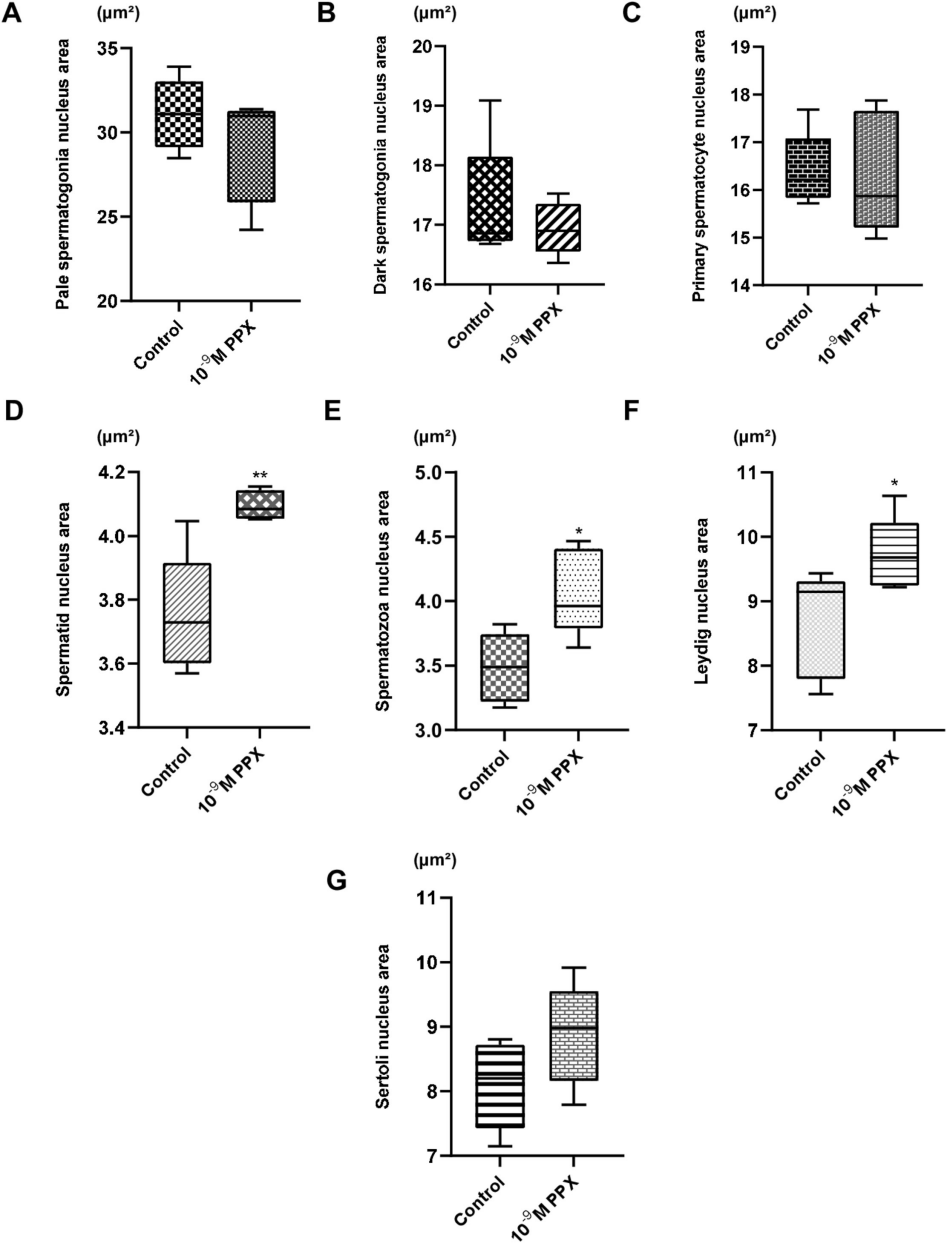
Effects of pyriproxyfen (10^−9^ M) exposure for 7 days on the germ cell nucleus area. Nucleus area values in pale spermatogonia (A) and dark spermatogonia (B); (C) primary spermatocytes, (D) spermatids, (E) spermatozoa, (F) Leydig cells, and (G) Sertoli cells. Data are expressed as means ± SEM of experiments with 5 fish in each group. * Indicates statistically significant (*p* ≤ 0.05) in relation to the control group, and ** indicates highly significant (*p* < 0.01), as determined by Student's *t*‐test for unpaired samples.

Figure 1A depicts an optical microscopy panorama of a control testis slice, highlighting different cysts in pale spermatogonia (ps), dark spermatogonia (ds), primary spermatocytes (spp), spermatid (spm), and spermatozoa (spz). Figure 1B presents the Sertoli cells (Sc). A comparison of Figure 1B with Figure 1C reveals an increased nuclear area in Leydig cells from testes treated with PPX. This may indicate that PPX interferes in the condensation of the chromatin of these cells, which have larger nuclei when compared with the control group. These data were statistically confirmed, as shown in Figure 2F. Furthermore, spermatid cells in the PPX‐treated group showed a significantly larger nuclear area compared with the control group, as showed in Figure 1D,E, with mean values increasing from 3.753 to 4.094 μm^2^ (Figure 2D). PPX did not significantly change the nucleus area in the pale and dark spermatogonia cells, as demonstrated in Figure 2A,B, respectively. Figure 2C shows that there was no significant effect of PPX on the nucleus area of the primary spermatocytes; in contrast, in the spermatozoa, the compound significantly increased the nucleus area, as shown in the control and treated cells (Figure 1D,E) and Figure 2E (with the mean area increasing from 3.484 to 4.071 μm^2^). The Leydig (Figures 1C and 2F) and Sertoli cells (Figures 1B and 2G), which do not participate directly in the cell division process of spermatogenesis but play crucial roles in testosterone production and cell nutrition, respectively, were analyzed. Only the Leydig cells were affected by PPX, demonstrating an increase in the area of the nucleus, when compared with the control group (with the mean area increasing from 8.674 to 9.722 μm^2^). The total cell area was not measured due to the difficulty in precisely observing and delineating cell boundaries under the experimental conditions. Therefore, it was not possible to determine whether the observed changes in nuclear area are independent of overall cell size. However, the results specifically indicate alterations in nuclear dimensions, suggesting a direct effect of PPX on nuclear morphology. Future studies incorporating precise cell size measurements would be necessary to fully elucidate the relationship between nuclear and cellular changes.

### 3.2 ADMET Prediction

This online tool provides predictive models for various physicochemical, pharmacokinetic, and toxicity parameters, facilitating the estimation of ADMET (Absorption, Distribution, Metabolism, Excretion and Toxicity) profiles for chemical compounds. Understanding the ADMET profile is essential for evaluating the biological activity and safety of the PPX compound, and key data are summarized in Table 1. PPX has a molecular weight of below 500 g/mol, a lipophilicity (Log P) ranging from 2.98 to 4.79, and fewer than four hydrogen bond donors. Its total polar surface area (TPSA) of approximately 40.58 Å^2^ is indicative of high gastrointestinal (GI) absorption. The Log P values suggest efficient absorption through biological membranes and a potential for crossing the blood–brain barrier (BBB), as supported by predictions from the SwissADME platform. However, PPX also exhibits significant genotoxicity and a propensity to induce drug‐related nephrotoxicity. Cytochrome P450 (CYP) isoenzymes play a critical role in the biotransformation of xenobiotics, and PPX notably inhibits CYP1A2, CYP2C19, CYP2C9, CYP2D6, and CYP3A4, which are key enzymes involved in drug metabolism. This inhibition may impair the metabolic clearance of other compounds, potentially leading to drug–drug interactions and toxicities (Lynch and Price 2007). These findings emphasize the need to account for CYP enzyme inhibition in the biotransformation and safety evaluation of PPX and related compounds, in further investigations.

**TABLE 1:** ADMET prediction data for pyriproxyfen. Topological polar surface area (TPSA); log of the partition coefficient from in‐house physics‐based method (iLOGP); log of the partition coefficient from atomistic and knowledge‐based method (XLOGP3); log of the partition coefficient from the topological method (MLOGP); hybrid fragmental/topological method (LOG P SILICOS‐IT); log of water solubility (LOG S); gastrointestinal (GI) absorption; brain–blood barrier (BBB) permeant; P‐glycoprotein (P‐gp) substrate; log of the skin permeability (Log Kp); cytochrome P450 (CYP).

Physicochemical properties		Lipophilicity LogP		Water solubility		ADME		Toxicity	
Molecular weight (g/mol)	321.37	iLOGP	3.67	Log S (ESOL)	−4.94	GI absorption	High	hERG blockers	0.735
00.Heavy atoms	24	XLOGP3	4.79	Class	Moderately soluble	BBB permeant	Yes	DILI	0.77
Aromatic heavy atoms	18	WLOGP	4.72	LOG S (Ali)	−5.37	P‐gp substrate	No	Carcinogenicity	0.894
Rotable bonds	7	MLOGP	2.98	Class	Moderately soluble	CYP1A2 inhibitor	Yes	Drug‐induced neurotoxicity	0.834
H‐bond acceptor	4	LOG P (SILICOS‐IT)	4.07	LOG S (SILICOS –IT)	−7.10	CYP2C19 inhibitor	Yes	Ototoxicity	0.571
H‐bond donor	0	Consensus	4.05	Class	Poorly soluble	CYP2C9 inhibitor	Yes	Eye irritation	0.486
TPSA (Å^2^)	40.58					CY3A4 inhibitor	Yes	Hematotoxicity	0.253
						CYP2D6 inhibitor	Yes	Drug‐induced nephrotoxicity	0.528
						Plasma protein binding	99.3%	AMES toxicity	0.638
						T1/2	0.622		

### 3.3 Homology Modeling and Structure Quality Assessment

**FIGURE 3:**
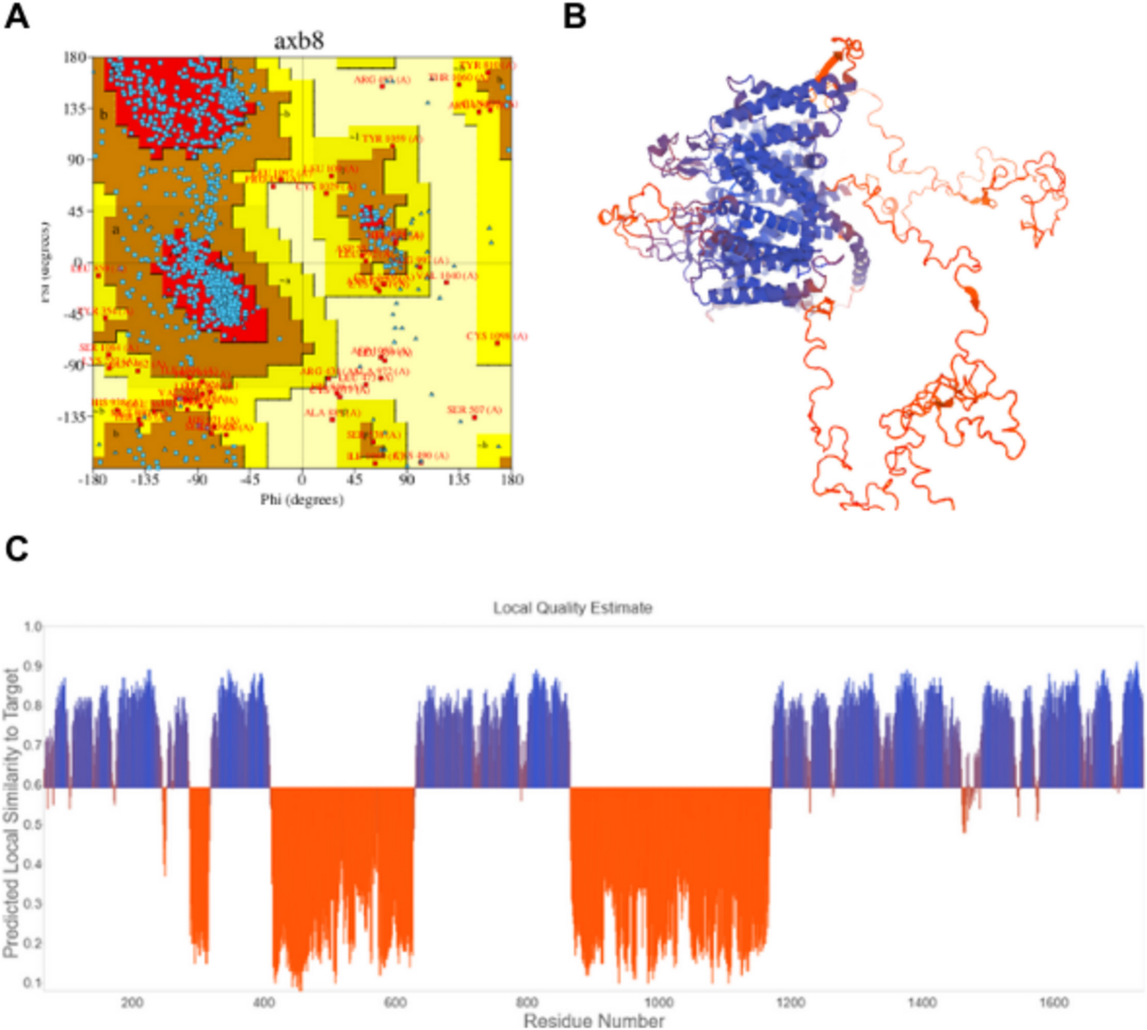
Ramachandran plot (A); Structural validation with a normalized QMEAN score of the theoretical 3D structure of TVDCC (B); Graphical representation of the amino acid residues corresponding to the 3D structure shown in figure B (C).

**FIGURE 4:**
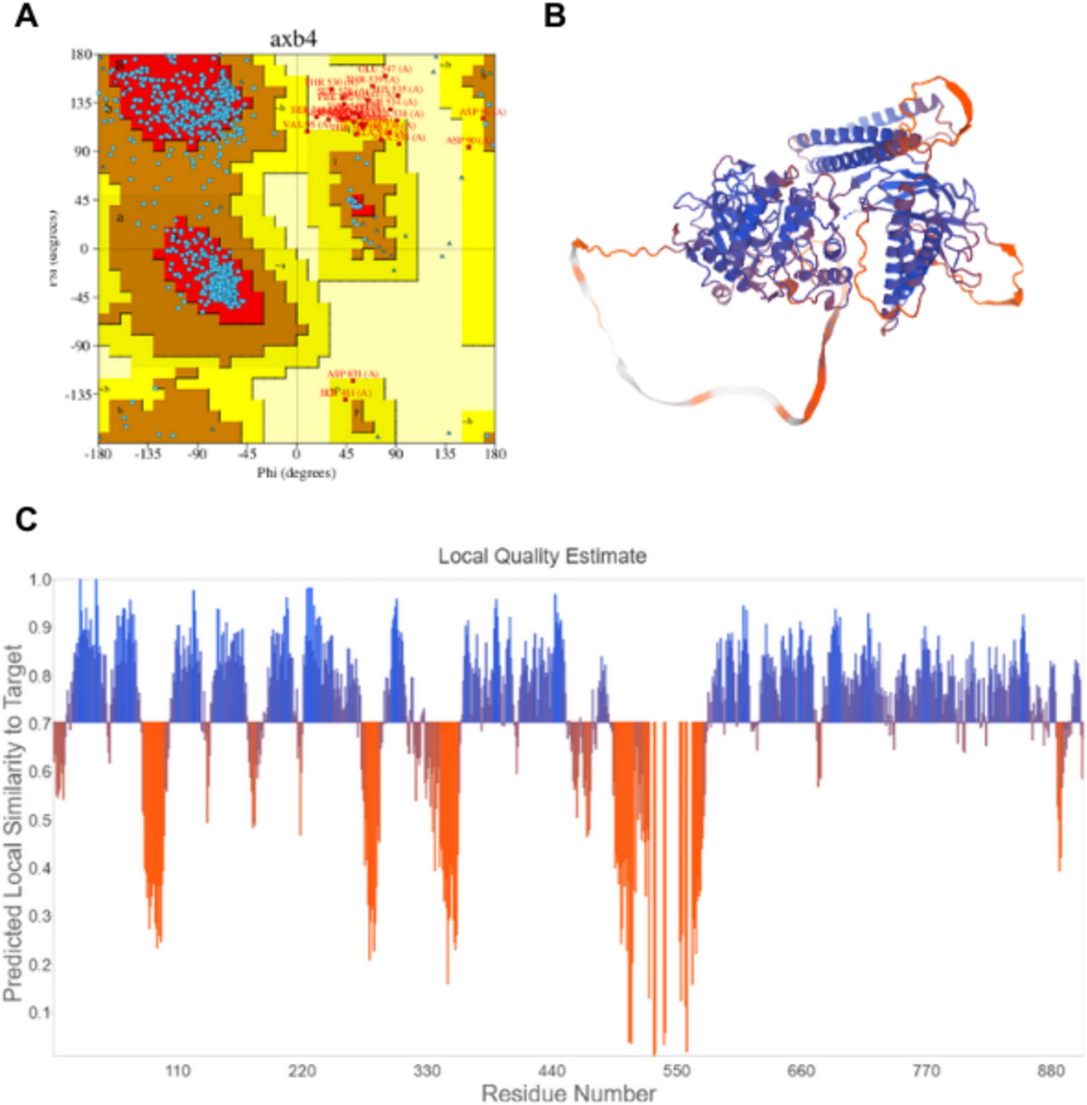
Ramachandran plot (A); Structural validation with a normalized QMEAN score of the theoretical 3D structure of PKC (B); Graphical representation of the amino acid residues corresponding to the 3D structure shown in figure B (C).

The T‐VDCC structure initially exhibited low quality due to the presence of undefined loops. Although structural modeling improved the overall quality, certain deficiencies persisted. However, focusing on the regions of interest where interactions with PPX occur, these areas are well represented, supporting the reliability of the method and experimental design. This is demonstrated in Figure 3A, which depicts the Ramachandran plot showing 81.7% of residues within highly favorable regions, indicative of higher structural quality. Moreover, the regions of interest (primarily residues 1607–1657) are highlighted in blue on the Qualitative Model Energy Analysis plot (QMEAN), corresponding to areas of greater confidence, as illustrated in Figure 3B,C. With regard to the PKC structure, although this is a predictive model that is generated by AlphaFold, it exhibited a higher overall quality compared with the T‐VDCC structure, with well‐preserved regions of interest. The Ramachandran plot revealed that 86.7% of residues fall within highly favorable regions, as shown in Figure 4A. Additionally, the QMEAN results further support the quality of the model and are presented in Figure 4B,C.

### 3.4 The Interaction of PPX With the T‐VDCC

The results of the molecular docking assay between pyriproxyfen and the T‐VDCC predicted binding poses with energies ranging from −6.60 to −8.99 kcal/mol (Table 2). These values reflect the stability of interactions between the molecule and the receptor, where more negative values indicate more favorable interactions and, consequently, higher affinity between the ligand and the target protein (Morris et al. 2009).

**TABLE 2:** Results of molecular docking performed for pyriproxyfen with the TVDDC by Autodock Vina/UCSF Chimera in version 1.17.3. Affinity energy, hydrophobic interactions, hydrogen bonds, and root mean square deviation (RMSD).

Ligand poses	Affinity energy (kcal/mol)		Hydrophobic interactions	Hydrogen bonds	RMSD
	Vina	Score only			
1	−8.880	−8.992	81.516	0.116	0.000
2	−8.212	−7.923	82.157	0.406	2.048
3	−8.034	−7.930	75.744	1.041	21.982
4	−7.963	−6.765	54.628	0.591	21.620
5	−7.956	−8.186	73.345	0.000	4.831
6	−7.857	−7.973	73.497	0.349	4.821
7	−7.732	−7.731	64.799	0.275	22.023
8	−7.724	−7.113	69.288	0.000	4.872
9	−7.670	−6.603	55.798	1.214	21.957

Binding energy values within this range are generally considered indicative of moderate to strong interactions. Comparatively, Neuro Molecular Products compounds (NMP‐4, NMP‐7, and NMP‐181), a class of molecules known as dual blockers of T‐type calcium channels and cannabinoid receptors, often exhibit average binding energies of −9.2 kcal/mol (Rangel‐Galván et al. 2022), a value close to that observed for pyriproxyfen.

**FIGURE 5:**
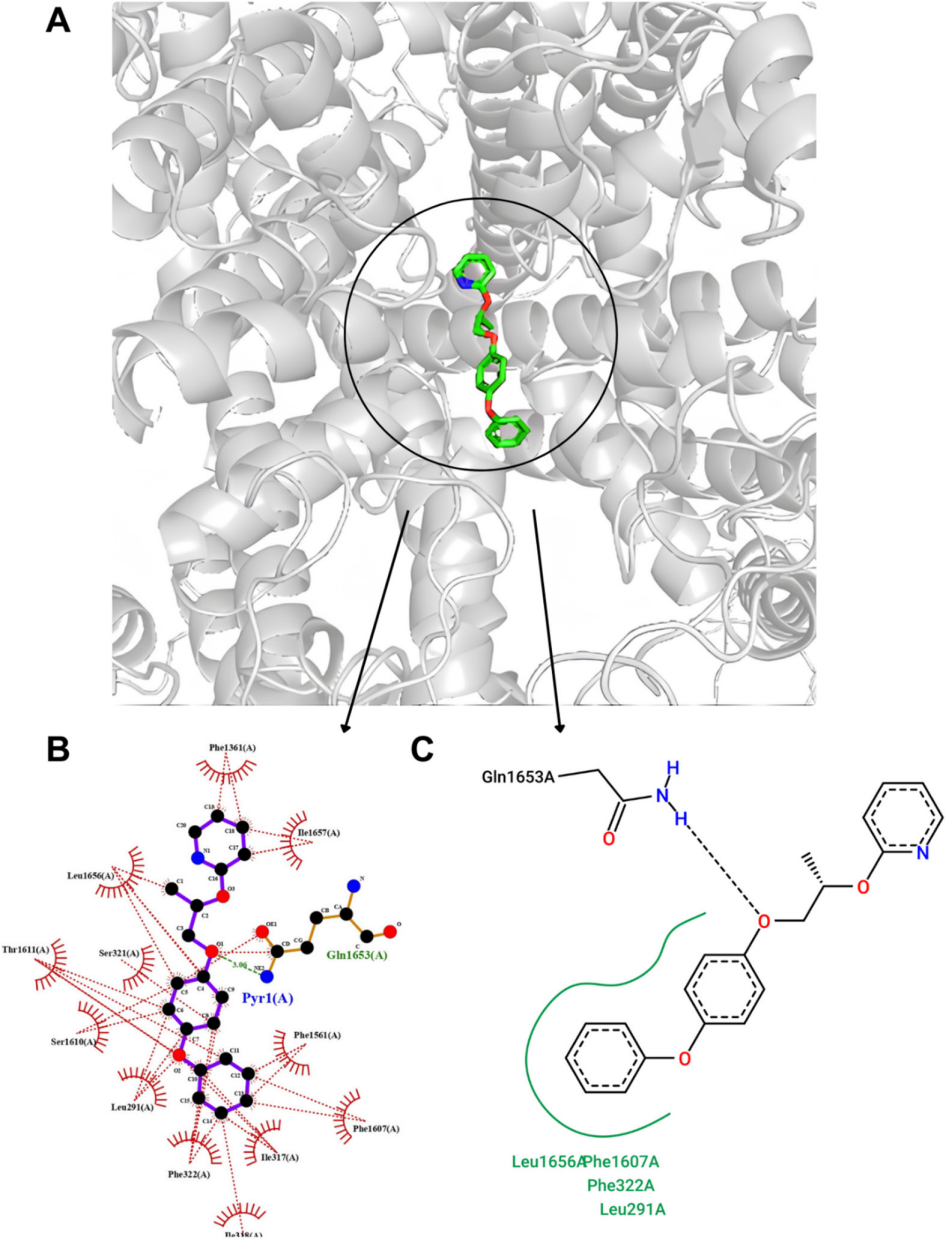
Representation of the molecular docking of pyriproxyfen with T‐type voltage‐dependent calcium channel (TVDCC; PDB ID: 7WLI) visualized in PyMOL (A); 2D interaction diagrams generated by LigPlot+ for pose 3 (B); PoseView for pose 3 (C) from ProteinsPlus.

These interactions include hydrogen bonds with the Gln1653 residue and hydrophobic interactions occurring primarily within hydrophobic pockets, commonly observed in protein–ligand interactions. Affinity energy, also referred to as binding free energy (Δ*G*), measures the strength and stability of protein–ligand interactions. This parameter is a key aspect of molecular docking, as it reflects the thermodynamic favorability of the binding process. Lower (more negative) Δ*G* values indicate stronger interactions, as they represent a spontaneous binding process in which the system achieves a lower energy state upon complex formation (Bissantz et al. 2010). Binding free energy is influenced by various factors, including hydrogen bonding, hydrophobic interactions, van der Waals forces, and electrostatic interactions. The key amino acid residues contributing to PPX binding are Leu291, Ile317, Ser321, Phe322, Phe1561, Phe1607, Thr1611, Gln1653, and Leu1656, as shown in Figure 5.

### 3.5 The Interaction of PPX With PKC

The molecular docking analysis predicted interactions between PPX and PKC, with binding energies as low as −8.36 kcal/mol, as reported in Table 3. In comparison, PMA, a natural PKC agonist, exhibits a binding energy of −3.2 kcal/mol (Gonzalez‐Guerrico and Kazanietz 2005).

**TABLE 3:** Results of molecular docking performed for pyriproxyfen with PKC by Autodock Vina/UCSF Chimera in version 1.17.3. Affinity energy, hydrophobic interactions, hydrogen bonds, and root mean square deviation (RMSD).

Ligand poses	Affinity energy (kcal/mol)		Hydrophobic interactions	Hydrogen bonds	RMSD
	Vina	Score only			
1	−7.900	−7.852	53.213	0.000	0.000
2	−7.700	−8.360	61.977	0.255	4.371
3	−7.500	−8.108	52.986	0.350	3.906
4	−7.400	−7.318	41.806	0.000	1.581
5	−7.300	−7.411	54.082	0.000	1.716
6	−7.100	−6.941	37.859	0.503	2.817
7	−7.100	−7.583	51.565	0.215	4.622
8	−7.000	−7.598	37.509	0.000	19.856
9	−7.000	−6.594	31.169	0.203	17.444

**FIGURE 6:**
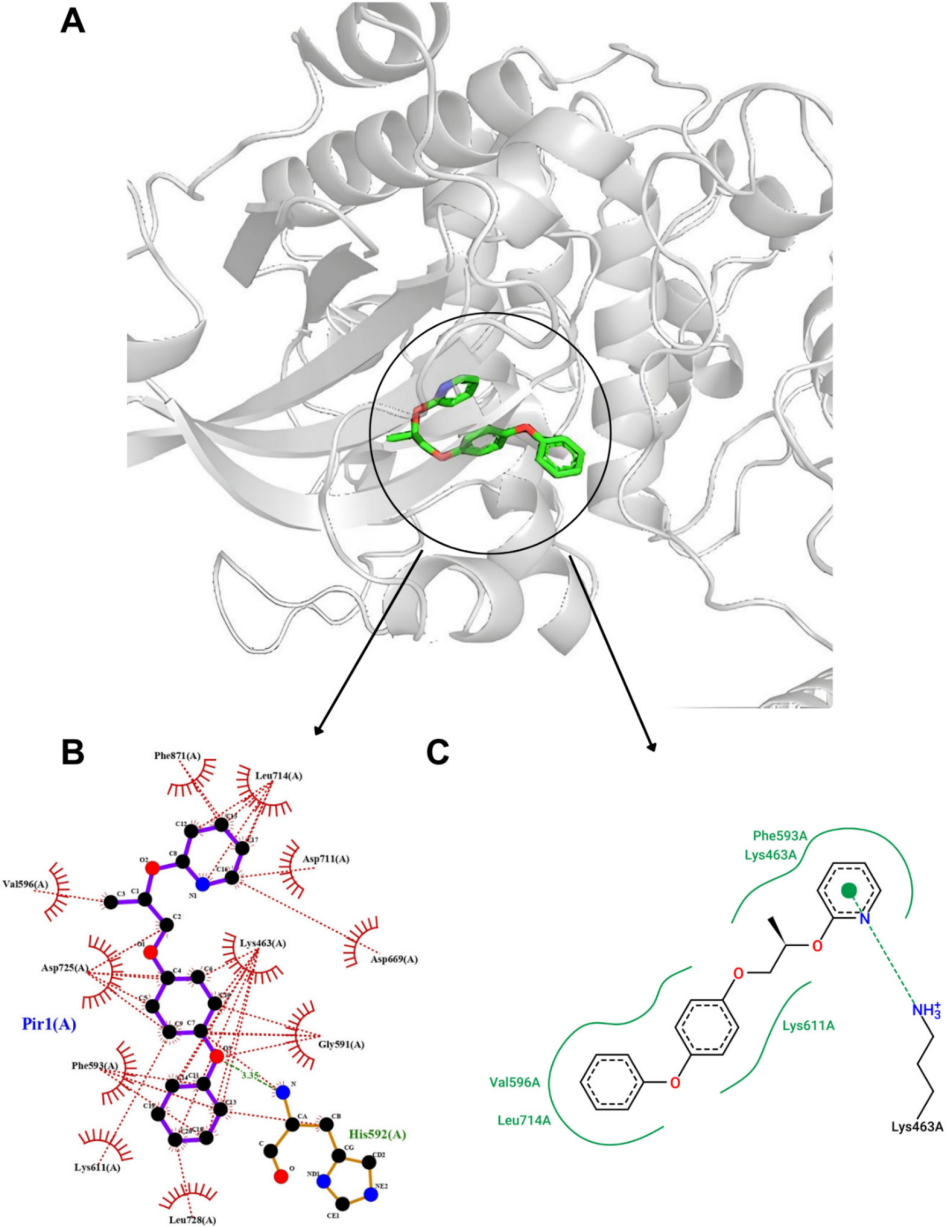
Representation of the molecular docking of pyriproxyfen with Protein Kinase C (PKC; AlphaFold code: AF‐A4QNX9‐F1‐v4) visualized in PyMOL (A); 2D interaction diagrams generated by LigPlot+ for pose 7 (B); PoseView for pose 3 (C) from ProteinsPlus.

The interactions between PPX and PKC include hydrophobic interactions, ionic bonding, and hydrogen bonds. The most notable interactions are ionic binding with Lys463, hydrogen bonds with the His592 residue (Figure 5A), and hydrophobic interactions involving Gly591, Val596, Lys611, Asp711, and Leu728, among others (Figure 6B,C).

## 4 Discussion

PPX is a widely used chemical that interferes with the male and female reproductive systems of the *D. rerio* (Oliveira et al. 2021, 2023; Silva et al. 2024), consistent with literature reports showing the adverse effects of PPX on the number of offspring of *D. magna* (Tatarazako et al. 2003). In addition, PPX interrupts spermatogenesis in seminiferous tubules of Swiss albino mice (Shahid et al. 2019) and alter the circulation of sexual hormones in *D. rerio* (Maharajan et al. 2018). Herein, PPX was found to alter the germ cell nucleus area (in spermatids and spermatozoa) in *D. rerio* , indicating that this pesticide may impair the condensation of chromatin in these cells, and potentially affecting male fertility. The reorganization of chromatin during spermatogenesis is a complex process. Initially, spermatozoa DNA is associated with histones, which are replaced by transition proteins and subsequently by protamines as spermiogenesis progresses. This change occurs before the spermatozoa exits the testes and results in a compacted spermatozoa chromatin that is resistant to denaturation, due to the disulfide bridging formed during transit in the epididymis (Rathke et al. 2014). Disruptions in transcriptional and translational processes can alter nucleoproteins, affecting the structure of spermatozoa chromatin. The integrity and stability of chromatin are directly related to male fertility and continuous embryonic development after fertilization. The integrity of chromatin also influences the expression of paternally inherited genes, potentially causing “male factor” infertility during embryonic development (D'occhio et al. 2007; Esteves et al. 2017).

Sperm morphology is a critical factor in the assessment of male fertility, as demonstrated in studies conducted on humans, because it is directly associated with reproductive potential and various functional sperm tests. Structural abnormalities and chromatin immaturity may be linked to infertility, as defects in sperm morphogenesis compromise nuclear DNA integrity and, consequently, sperm functionality (Franken 2015; Jakubik‐Uljasz et al. 2020). Further research on sperm morphological alterations and infertility in *D. rerio* is essential to deepen our understanding of reproductive biology and its implications for both aquatic species and human health.

Furthermore, the testicular somatic cells responsible for the synthesis and secretion of testosterone, the Leydig cells, exhibited an augmented nucleus area after short‐term treatment (7 days) even treated with low pesticide concentration (10^−9^ M PPX, i.e., 30× lower than that recommended by health agencies to be included in the potable water) (WHO 2007; WHOPES 2020). This alteration in morphology may be related to the DNA transcription and the regulation of key enzymes (*StAR*, *CYP11A1*, *17β‐HSD*, and *CYP17A1*) involved in the complex progression of steroidogenesis (Carreau et al. 2011; Lee et al. 2024). The incidence of defects in steroidogenesis may also result in impaired sexual development (Singh and Singh 2019; Widhiantara et al. 2021). In a whole, our data points that the chromatin, biotransformation enzymes, and steroidogenesis enzymes may be relevant targets to PPX mediated by calcium channels activity and PKC. So, it deserves additional studies.

The T‐VDCC is present in multiple cell types, directly influencing their functions and maturation by stimulating signal transduction, a process also observed in cells involved in spermatogenesis (Darszon and Hernández‐Cruz 2014). Affinity energy serves as an indicator of the intensity of the interaction between the compound and the protein, with an inverse relationship. In the present study, PPX was found to interact with the T‐VDCC primarily through hydrophobic interactions and hydrogen bonding, which could potentially trigger a response, leading to the opening or closing of the channel. This, in turn, may lead to a PPX‐induced increase in intracellular calcium, as discussed by Oliveira et al. (2021).

PKC is a protein present in the testicular cells of *D. rerio* and in rats. This kinase participates in bisphenol A and 1,25(OH)_2_ vitamin D_3_ signaling (Batista‐Silva et al. 2020b; Gonçalves et al. 2017) by modulating calcium influx, the phosphorylation of important proteins, and the gene expression of enzymes (Tan and Parker 2003). The ATP‐binding site of PKC is located between amino acid residues 357–364 in the human protein, as indicated in Uniprot (Holliday et al. 2015). This sequence is similar to that of the amino acid residues 588 to 596 in *D. rerio* , identified using the MatchMaker and MatchAlign tools available in UCSF Chimera, where some interactions with PPX occur.

The interaction of PPX with this protein occurs in the region where PKC may be phosphorylated by the ATP molecule (residues 588–596). Therefore, PPX may be a competitor for this site, preventing PKC from being phosphorylated, and consequently inhibiting the phosphorylation of other proteins, thereby disrupting downstream signaling. The interaction between the pesticide and the target protein occurs at a favorable energy level and involves various binding interactions, all of which are strong and significant, as demonstrated by the in silico study.

Calcium is involved in the regulation of chromatin condensation, a vital process for forming a compact hydrodynamic shape in spermatozoa (Golpour et al. 2017). Chromatin condensation provides protection against physical and chemical damage to the parental genome and influences epigenetic regulations mediated by protamines (Martin and Cardoso 2010). In specific stages, such as in spermatids and *D. rerio* spermatozoa, calcium forms unbound pools in the nucleus, suggesting a role in the regulation of chromatin condensation and compaction (Golpour et al. 2017). This complex interaction between calcium and spermatogenesis is fundamental for the suitable development of spermatozoa and, therefore, for fertility and reproduction. However, it should be taken into consideration that substances like PPX, present in the environment, may interfere with this process, potentially leading to adverse changes in development and fertility.

## 5 Conclusion

Following our previous studies, we investigated the ex vivo short‐term effects of PPX on the testes of adult *D. rerio* . The insecticide was found to alter the nuclear area of key cells involved in spermatogenesis (spermatids and spermatozoa) as well as steroidogenesis (Leydig cells). ADMET and molecular docking studies further corroborated these findings, revealing that PPX is sufficiently lipophilic to permeate cell membranes. Additionally, the compound exhibited potential strong binding affinities for both T‐VDCCs and PKC. These interactions may suggest a potential effect of pyriproxyfen on the modulation of T‐VDCC and PKC function, which could be relevant for future investigations into its physiological and toxicological impact. These data suggest that short‐term exposure to PPX disrupts the morphology of spermatogenic and steroidogenic cells, unveiling a bimodal interface between morphology and molecular targets for PPX on signaling pathways, which could impair male reproductive health or lead to infertility. Additional studies, such as molecular dynamics simulations and experimental assays, are necessary to validate these findings and determine the actual impact of the observed interactions.

## Author Contributions

Conceptualization: FRMBS and RCP; data curation: FRMBS, RCP, GAAL, and VMASG; formal analysis: FRMBS, BAZ, VSO, and VMASG; funding acquisition: FRMBS; investigation: BAZ, VSO, and GAAL; methodology: BAZ, VSO, GAAL, and VMASG; project administration: FRMBS and RCP; resources: FRMBS and RCP; software: RCP, VMASG, and GAAL; supervision: FRMBS and RCP; validation: RCP, FRMBS, and GAAL; visualization: FRMBS, GAAL, and RCP; writing – original draft: BAZ, VMASG, and GAAL; writing – review and editing: FRMBS, RCP, VMASG, VSO, BAZ, and GAAL. All authors revised the final version and agreed to this submission.

## Conflicts of Interest

The authors declare no conflicts of interest.

## Data Availability

The data that support the findings of this study are available on request from the corresponding author. The data are not publicly available due to privacy or ethical restrictions.
